# Evidence of a Role for the Lateral Hypothalamic Area Juxtadorsomedial Region (LHAjd) in Defensive Behaviors Associated with Social Defeat

**DOI:** 10.3389/fnsys.2016.00092

**Published:** 2016-11-14

**Authors:** Miguel J. Rangel, Marcus V. C. Baldo, Newton S. Canteras, Joel D. Hahn

**Affiliations:** ^1^Department of Anatomy, University of São PauloSão Paulo, Brazil; ^2^Department of Physiology and Biophysics, University of São PauloSão Paulo, Brazil; ^3^Department of Biological Sciences, University of Southern CaliforniaLos Angeles, CA, USA

**Keywords:** LHAjd, social defeat, hypothalamus, lateral hypothalamic area, behavior, animal

## Abstract

Our understanding of the extrinsic connections of the lateral hypothalamic area (LHA) has deepened in recent years. In particular, a series of studies using neural pathway-tracing methods to investigate the macroconnections of histologically differentiated LHA regions, have revealed that the neural connections of these regions are substantially distinct, and have robust connections with neural circuits controlling survival behaviors. To begin testing functional associations suggested by the distinct LHA region neural connections, the present study has investigated the role of the LHA juxtadorsomedial region (LHAjd) in the control of social defeat (a socially-relevant defensive behavior). Male rats received bilateral cytotoxic lesions targeted to the LHAjd. A resident-intruder paradigm was then employed to investigate the effect of these lesions on defensive behavioral responses. Behavioral data were collected during three phases of testing: (1) pre-encounter habituation to testing context; (2) encounter with a dominant conspecific in the testing context; and (3) post-encounter context. Statistical analysis of behavioral measures revealed a significant decrease in risk assessment behaviors during post-encounter context testing in lesioned intruders compared to sham-lesioned and intact rats. However, changes in defensive behavioral measures during the habituation, or during resident-intruder encounters, did not reach significance. We discuss these data in relation to LHAjd (and neighboring LHA region) neural connections, and in relation to current advances in understanding of the neural control of defensive behaviors. A refined model for the neural circuits that are central to the control of socially-relevant defensive behaviors is outlined. We also consider possible broader implications of these data for disorders of behavioral control.

## Introduction

In rats, as in other animals, social defeat can occur as a consequence of social conflict with a dominant aggressor (Blanchard et al., 1977). In this scenario, a sequence of stereotyped behavioral interactions occurs, leading ultimately to behavioral expressions of social defeat in the subordinate animal (Blanchard et al., 1977). In addition to the expression of innate defensive behavioral responses occurring at the time of a stressful social conflict and defeat, socially defeated animals also typically express post-conflict defensive behaviors in the absence of the original stressor that are elicited by learned conflict-associated contextual cues (Motta et al., 2009; Faturi et al., 2014).

The hypothalamus plays a critical role in the control of survival behaviors (for reviews see Risold et al., 1997; Swanson, 2000), and our understanding of the organization and relations of hypothalamic neural circuits underlying behavioral responses to stressful stimuli has increased markedly in recent decades (Simerly and Swanson, 1988; Canteras and Swanson, 1992a,b; Canteras et al., 1992a,b, 1994, 1995, 1997; Risold et al., 1994; Chiavegatto et al., 1998; Canteras and Goto, 1999). Over the past decade, attention has refocused on the spatially-extensive and poorly understood lateral hypothalamic area (LHA), leading to a series of high spatial resolution neural pathway-tracing studies that have determined the extrinsic macroconnections of the LHA medial- and perifornical tiers (Goto et al., 2001, 2005; Goto and Swanson, 2004; Hahn and Swanson, 2010, 2012, 2015). Among the numerous connections identified, it emerged that two neighboring LHA medial tier regions (the LHA juxtaparaventricular—LHAjp, and especially the LHA juxtadorsomedial—LHAjd) are connected to a previously identified social threat responsive hypothalamic circuit involving the medial preoptic area (MPO), medial preoptic nucleus (MPN—embedded within the MPO), ventromedial hypothalamic nucleus (VMHvl; ventrolateral part), tuberal nucleus (TU) and the ventral premammillary nucleus (PMv; Motta et al., 2009; Hahn and Swanson, 2012; Faturi et al., 2014). The LHAjd and LHAjp also have robust connections with the dorsal premammillary nucleus (PMd; in particular, they provide an input to a dorsomedial subregion of the PMd; Hahn and Swanson, 2010, 2012) that is indicated to play a critical role in innate defensive responses (Canteras et al., 1997, 2008; Markham et al., 2004; Blanchard et al., 2005; Cezario et al., 2008; Motta et al., 2009).

These correlated data coalesced in two recent articles: the first of these reported that socially-defeated rats had a robust increase in the expression of the immediate early gene product cFos in the LHAjd after exposure to a social defeat-associated context (Faturi et al., 2014); more recently, a significant increase in LHAjd and LHAjp cFos was reported to occur in response to the social defeat itself, and also to the stress of entrapped immobilization (Motta and Canteras, 2015). Taken together, these data suggest a role for the LHAjd (and LHAjp) in both learned and innate defensive behavioral responses to more than one type of threat. Given the accumulating neuroanatomical and neuroactivational evidence implicating the LHAjd in the control of innate and learned defensive behavioral responses, notably in relation to socially-relevant fear-associated stimuli, in the present study we have investigated this possibility further with a series of experiments employing a territorial resident-intruder paradigm and cytotoxic lesions targeted to the LHAjd.

## Materials and Methods

### Animals

Animals were maintained in accordance with the guidelines of the Brazilian Association for Laboratory Animal Science (Sociedade Brasileira de Ciência em Animais de Laboratório; COBEA), and the Guide for the Care and Use of Laboratory Animals (National Research Council, USA, 2011). In addition, all experimental procedures involving animals were approved by the Committee on Care and Use of Laboratory Animals of the Institute of Biomedical Sciences, University of São Paulo, Brazil (Instituto de Ciências Biomédicas, Universidade de São Paulo; Protocol number 130/2005). For resident-intruder behavioral experiments, male Wistar rats (*n* = 32, 3–4 months old, approximately 300 g) were used as intruders; Long Evans males (*n* = 4, 9–12 months old, approximately 600 g) were used as residents (the latter were housed with Long Evans female rats, *n* = 4, 3–5 months old, approximately 300 g). Long Evans rats are commonly used as residents in a resident-intruder paradigm because they display high levels of aggression toward young male conspecifics (Thor and Flannelly, 1976). All animals were obtained from local breeding facilities, and were housed in dedicated animal housing facilities under controlled temperature (23°C) and illumination (12/12-h light/dark cycle), and with unrestricted access to food (standard laboratory diet) and water.

### LHAjd NMDA and Sham Lesions

Male Wistar rats (*n* = 20) were deeply anesthetized with sodium pentobarbital (40 mg/kg, IP; Cristália: Itapira, SP, Brazil) and received bilateral *N*-Methyl-D-aspartate (NMDA; 0.15 M) injections targeted stereotaxically to the LHAjd (typical coordinates: 2.2 mm caudal to bregma, 0.8 mm lateral to the middle of the superior sagittal sinus, 7.8 mm ventral to surface of cerebral cortex). The NMDA was injected iontophoretically from glass micropipettes (approximate tip diameter 20 μm) using a constant current source (Model CS3, Midgard Electronics) with the following parameters: 10 μA (negative polarity), 7 s current on/off, 10 min/side. Additional rats (control groups, *n* = 12) either received saline injections (sham lesion, *n* = 5), or were not injected (intact; *n* = 7). Rats were allowed a 2-week post-surgery recovery period before they were used in resident-intruder behavioral testing.

### Resident-Intruder Behavioral Experiments

Methods for the resident-intruder behavioral experiments followed those described previously (Ribeiro-Barbosa et al., 2005; Faturi et al., 2014). Wistar rats were housed individually; male and female Long Evans rats were housed together in pairs for 3 weeks prior to use of the Long Evans males as residents in behavioral testing. Two weeks prior to pairing, the Long Evans females were sterilized by severing their uterine horns (partial hysterectomy), to prevent pregnancy while retaining ovarian function and sexual behavior—this surgical procedure was performed under deep anesthesia (mixture of ketamine and xylazine; 1:2 v/v; 1 ml/kg body weight). Animals were housed in transparent acrylic (Plexiglas) home cages (25 cm cube with a 12.5 cm width vertically sliding access panel positioned centrally on one side). All behavioral experiments were video recorded for subsequent analysis.

#### Habituation to Context

For 10 days, each Wistar rat (NMDA lesion, sham lesion, or intact) was isolated in its home cage. At the beginning of the light phase the rat was transferred in its home cage from a housing room to an adjacent procedure room. The home cage access panel was then raised for 10 min, allowing egress and free exploration of an enclosed Plexiglas corridor (100 cm length × 30 cm height × 12.5 cm width) and (at the other end of the corridor) a second cage of identical construction to the home cage, into which were placed food pellets the rat could obtain. A small amount of fresh bedding was placed in the testing apparatus (corridor and second cage) prior to habituation. After the 10-min habituation period the rat was returned in its home cage to the housing room. The corridor and second cage of the apparatus were cleaned between each habituation session.

#### Resident-Intruder Encounter

After 10 days of habituation to context, on the next day, the second cage (food compartment) was replaced with the Long Evans pair resident home cage (with the female removed for the duration of the encounter). The Wistar male intruder was allowed access to the resident home cage following the habituation protocol of the prior 10 days, and once inside the resident’s cage, the access panel was lowered to prevent egress. Only experienced resident males were used for resident-intruder encounters. If a clear attack (bite) occurred within the first 10 min of an encounter, the resident and intruder were allowed to remain together for a further 10 min after the first attack; if an attack did not occur in the first 10 min, the pair were separated (and these intruders were excluded from subsequent testing and analysis, *n* = 1 from the experimental group, none from control groups).

#### Post-Encounter Context

On the day after an encounter and social defeat, socially-defeated intruders were allowed to explore the testing context for 5 min. That is, a shortened version of the habituation protocol was followed, with the resident’s home cage placed at the other end of the connecting corridor, and with the resident removed from its home cage for the duration of the experiment.

### Histology

Ninety (90) minutes after the start of the post-encounter context testing, rats were deeply anesthetized (sodium pentobarbital 40 mg/kg, IP), and then perfused transcardially with ice-cooled 0.9% saline, followed by ice-cooled 4% paraformaldehyde in 0.1 M phosphate buffer pH 7.4. The perfusion-fixed brains were removed and placed overnight in a solution of 20% sucrose in 0.1M phosphate buffer pH 7.4 at 4°C. They were then frozen on dry-ice and sectioned on a sliding microtome in the transverse (coronal) plane into four stepwise collated series (40 μM thickness). One of the series was processed for detection of Nissl substance (thionine stain) to confirm cannulae placement and cytotoxic lesion extent. For additional analysis of the lesions a second series of sections was processed for immunohistochemical (IHC) detection of NeuN (Anti-NeuN, MAB377, clone A60, Millipore, USA); the remaining series of sections were transferred to an anti-freeze solution and stored at −20°C for future use. For NeuN IHC, (in brief) the sections were incubated overnight in primary antibody (1:1000 dilution), then for 90 min at room temperature in a solution of biotinylated goat anti-mouse IgG (Vector Laboratories, Burlingame, CA, USA; 1:200 dilution). The sections were then exposed to an avidin–biotin horseradish peroxidase (HRP) reagent (ABC Elite Kit; Vector Laboratories) for 90 min. To visualize the location of the bound NeuN antibodies, the sections were exposed for 10-min to a solution containing 0.02% of a chromogen (3,3-diaminobenzidine tetrahydrochloride—DAB; Sigma, St Louis, MO, USA) and 0.3% nickel–ammonium sulfate in 0.05 M Tris–buffer (pH 7.6), followed by the addition of hydrogen peroxide (1:3000 dilution) and a further 10 min incubation, resulting in a dark blue-black product. The reaction was stopped by extensive washing in potassium phosphate-buffered saline pH 7.4 (KPBS). Sections were mounted on gelatin-coated slides, air-dried, dehydrated through an ascending series of alcohols, cleared with xylene, and coverslipped with DePeX (Sigma). During antibody incubation steps sections were refrigerated; antibodies were diluted in KPBS, that was also used for multiple washes between the incubation steps.

### Data Quantification

Data were quantified cumulatively for the intruder for a period sufficient for quantification for the three phases of the experiment (habituation, encounter and context re-exposure). Measurements for the first 5 min of habituation to context, and 5-min post-encounter context testing, included: (1) spatiotemporal measurements: time spent in home cage, corridor and second cage; and (2) duration of the following behaviors: risk assessment, exploration, rearing and grooming. Measurements for 10-min of resident-intruder agonistic encounters included the duration of following behaviors: passive defense, active defense, locomotion, grooming and social investigation. Data quantification (behavioral scoring) from video recordings was done by a trained observer using dedicated analysis software (The Observer, version XT; Noldus, Netherlands). Only intruders that had suffered a clear social defeat were used in the present analysis. The criteria used for scoring encoded behavioral measures are as follows:

- Risk assessment: (1) crouch-sniff (animal immobile with its back arched but actively sniffing and scanning the environment); and (2) stretch postures (animal’s body stretched forward, either motionless or moving slowly toward the second/resident cage).
- Exploration: (1) fearless locomotion (locomotion with arched back); and (2) upright position (animal actively exploring the environment, standing over its rear paws and leaning on the wall with the fore paws).
- Rearing: animal standing over its rear paws without wall contact.
- Grooming: self-cleaning behavior.
- Social investigation: intruder animal sniffing and making light exploratory paw contact with the body of the resident animal.
- Locomotion: forward movement.
- Passive defense: animal motionless and supine (on-the-back submissive posture).
- Active defense: including (1) intruder animal pushing away the resident animal; (2) assuming an upright position with sparse boxing; and (3) attempts to flee from the resident.

### Statistical Analysis

After testing for homogeneity of variance (Levene’s test), the behavioral data were analyzed using a parametric a univariate analysis of variance (ANOVA) for each dependent variable followed by a *post hoc* analysis using Tukey’s honest significant difference (HSD) test (*α* = 0.05) to isolate respective effects. Due to the number of dependent variables, we applied the Bonferroni’s correction to the significance level in the ANOVA (*α* = 0.007 for the behavioral measurements during the last day of habituation and during exposure to the context associated with social defeat; *α* = 0.01 for behavioral measurements associated with social defeat during encounter with aggressive conspecific).

## Results

**Figure 1:**
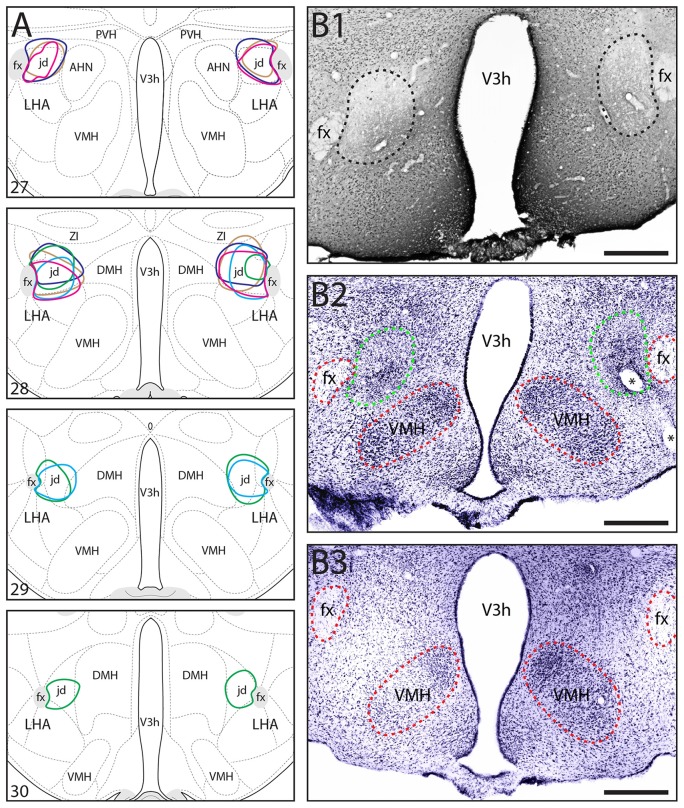
**(A)** Location and extent of bilateral *N*-Methyl-D-aspartate (NMDA) lesions including the lateral hypothalamic area (LHA) LHAjd in five socially-defeated rats that were used for behavioral analysis. The approximate location and extent of each lesion was determined by analysis of Nissl-stained cytoarchitecture (each is indicated by a different color). For comparison, the data are plotted on a reference rat brain atlas (numbered atlas levels are indicated; Swanson, 2004). **(B1,B2)** Representative digital photomicrographs of NeuN-labeled **(B1)**, and Nissl-stained **(B2)** cytoarchitecture indicating the general region of a bilateral NMDA lesion including the LHAjd (corresponds to region indicated by light blue polygon in **A**); **(B3)** shows the Nissl-stain for a sham-lesioned (vehicle injected) control at a similar rostro-caudal level. The approximate boundary of the lesion in **(B1,B2)** is indicated by a dashed line (black in **B1** green in **B2**); red dashed lines indicate additional fiducial markers (asterisks in **B2** indicate the location of a blood vessel). Images adjusted for brightness/contrast, Nissl images pseudocolored from grayscale. Abbreviations: AHN, anterior hypothalamic nucleus; DMH, dorsomedial hypothalamic nucleus; fx, fornix; LHAjd, lateral hypothalamic area, juxtadorsomedial region; PVH, paraventricular nucleus of the hypothalamus; V3h, third ventricle, hypothalamic part; VMH, ventromedial hypothalamic nucleus; ZI, zona incerta. Scale bars = 200 μm.

For the rats that received NMDA lesions targeted to the LHAjd, only those with bilateral lesions substantially restricted to the LHAjd were included in the present analysis (*n* = 5; Figure 1). Spatiotemporal and behavioral measurements were taken on the last day of habituation to the testing environment, on the next day during encounter with the conspecific aggressor, and again on the day following the encounter and social defeat. Over the first few days of the habituation, risk assessment behaviors were observed frequently; however, by day 3 (typically) these behaviors diminished, and by the end of the habituation phase (day 10) were not observed. These observations concur with the results of a recent study using the same experimental paradigm (Faturi et al., 2014). As shown in Table 1, during exposure to the testing environment on the last day of habituation, the ANOVA revealed no significant differences among the groups for either the spatiotemporal (*F*_(2,14)_ < 2.82, *p* > 0.093) or behavioral (*F*_(2,14)_ < 2.83, *p* > 0.092) measurements.

**Table 1:** **Spatiotemporal and behavioral measurements during habituation to context (10th day)**.

	Experimental groups			
	Intact (*n* = 7)	LHAjd lesion (*n* = 5)	Sham lesion (*n* = 5)	Statistics (*F*_(2,14)_, *p*)
**Spatiotemporal measurements**	
Home cage	61.4 ± 13.7	23.4 ± 10.6	55, 4 ± 10.5	2.82, 0.093
Corridor	86.1 ± 9.5	107 ± 12.5	112.8 ± 14.7	1.39, 0.280
Resident cage	152.7 ± 12.6	169.8 ± 20	132 ± 7.7	1.5, 0.255
**Behavioral items measured**	
Risk assessment	3.3 ± 1.6	12.4 ± 6.5	11.2 ± 5	2.83, 0.092
Exploration	274.2 ± 9.9	271.8 ± 5.4	281.2 ± 6.3	0.3, 0.743
Rearing	3 ± 1.1	3.4 ± 1.4	3 ± 3	0.34, 0.712
Grooming	20 ± 10.4	12.4 ± 5.4	4.4 ± 3.4	0.92, 0.422

*Values are mean ± SEM of the time in seconds during a 5-min observation period*.

Behavioral interactions during the resident-intruder encounter were comparable to those described previously: a typically short (<30 s) latency to first attack by the dominant aggressor (resident), and predominantly passive subordinate (intruder) defensive responses (remaining mainly motionless; Faturi et al., 2014). The ANOVA revealed no significant differences among the groups for the behavioral measurements (*F*_(2,14)_ < 3.31, *p* = 0.066; Table 2). However, during exposure to the social defeat-associated context, the ANOVA revealed a significant main effect for risk assessment measurements (*F*_(2,14)_ = 10.16, *p* = 0.0018). Moreover, in animals that received bilateral LHAjd lesions, there appeared to be an increase in the time of fearless exploratory locomotion (*F*_(2,14)_ = 3.92, *p* = 0.044; Table 3), but after applying Bonferroni’s correction (*α* = 0.007) this behavioral measurement did not differ significantly among the groups. For the other spatiotemporal and behavioral measurements, the ANOVA revealed no differences among the groups during exposure to the social defeat-associated context (Table 3).

**Table 2:** **Behavioral measurements during encounter (11th day)**.

	Experimental groups			
	Intact (*n* = 7)	LHAjd lesion (*n* = 5)	Sham lesion (*n* = 5)	Statistics (*F*_(2,14)_, *p*)
**Behavioral items**
Passive defense	525.5 ± 20.9	360.6 ± 82.4	438.6 ± 53.4	2.73, 0.099
Active defense	58.5 ± 19.5	182.0 ± 50.8	120.8 ± 39.5	3.31, 0.066
Locomotion	11.9 ± 4.37	17.8 ± 13.8	21.1 ± 11.1	0.23, 0.791
Grooming	0.2 ± 0.2	4.0 ± 2.8	8.7 ± 4.2	1.7, 0.217
Social investigation	3.8 ± 3.8	35.4 ± 23.8	10.4 ± 4.8	1.41, 0.275

*Values are mean ± SEM of the time in seconds during a 10-min observation period*.

**Table 3:** **Behavioral measurements during context re-exposure after social defeat (12th day)**.

	Experimental groups			
	Intact (*n* = 7)	LHAjd lesion (*n* = 5)	Sham lesion (*n* = 5)	Statistics (*F*_(2,14)_, *p*)
**Spatiotemporal measurements**	
Home cage	98.1 ± 29.7	101.0 ± 19.9	114.8 ± 47.8	0.06, 0.93
Corridor	154.9 ± 29.1	94.4 ± 15.6	107.8 ± 32.8	1.08, 0.364
Resident cage	47.0 ± 25.7	104.8 ± 29.9	77.6 ± 20.7	1.6, 0.234
**Behavioral items**	
Risk assessment	180.9 ± 7.7	81.0 ± 8.9*	182.0 ± 38.3	10.16, 0.0018
Exploration	107.3 ± 10.4	207.4 ± 7.0	111.2 ± 36.2	3.915, 0.044
Rearing	0.6 ± 0.3	1.8 ± 0.8	1.2 ± 0.4	1.02, 0.382
Grooming	9.9 ± 4.4	9.6 ± 5.1	4.6 ± 2.0	0.40, 0.677

Behavioral measurements during encounter (11th day). Values are mean ± SEM of the time in seconds during a 5-min observation period. *Differs significantly from sham-lesioned and intact control groups (*p* < 0.01, Tukey HSD test).

**Figure 2:**
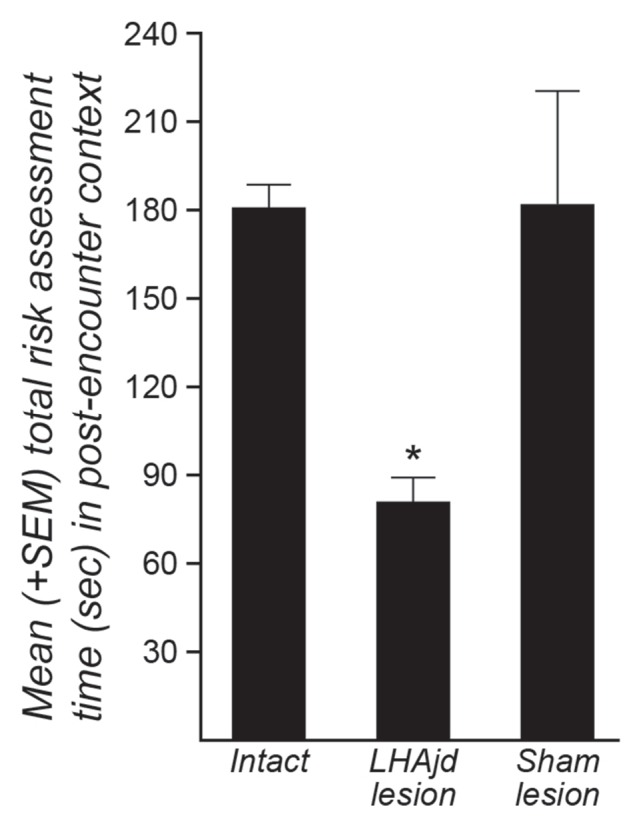
**Mean (+SEM) risk assessment behavior duration for three groups of rats (intact, *n* = 7; bilateral NMDA LHAjd lesion *n* = 5; bilateral [vehicle injected] sham lesion *n* = 5) quantified for a 5-min period during exposure of socially-defeated intruders to the environmental context in which they experienced a social defeat on the previous day.** (*p = 0.0018).

*Post hoc* pairwise comparison revealed for the animals exposed to the social defeat-associated context that bilateral LHAjd lesions significantly decreased risk assessment responses compared to the other experimental groups (*p* < 0.007, Tukey’s HSD test; see also Table 3; Figure 2). Overall, the present results suggest that bilateral LHAjd lesions do not have a significant impact on innate social defensive behavioral responses, but do significantly impact contextual responses, in particular risk assessment behavior in the environmental context associated previously with a stressful social defeat event.

## Discussion

The experimental paradigm used in the present study was established in a recent study that showed rats exposed to a single social defeat event, and then re-exposed to the defeat-associated context, displayed robust and reproducible defensive behavioral responses (Faturi et al., 2014). In the previous study it was also reported that the socially-defeated animals had a marked increase in their levels of LHAjd cFos expression compared to controls, and further that muscimol blockade of GABA_A_ receptors in either the PMd or the dorsal division of the periaqueductal gray (PAGd) immediately prior to re-exposing them to the defeat-associated context, resulted in a significant attenuation of risk assessment behavior (Faturi et al., 2014). Here we have reported a comparable significant attenuation of risk assessment behavior resulting from cytotoxic (NMDA) lesion of LHAjd neurons that provide a moderately robust input to the PAGd, and a very robust input to the PMd (Hahn and Swanson, 2012).

The PMd and PAGd are both extensively characterized key nodes for the control of defensive behavioral responses to different types of threat stimuli (Cezario et al., 2008; Motta et al., 2009; Sukikara et al., 2010; Motta and Canteras, 2015). Thus, like the LHAjd, the PMd and PAGd both show increased expression of cFos in rats re-exposed to the context of a social defeat (Faturi et al., 2014). Moreover, increased PMd and PAGd cFos expression is also seen directly after social defeat, and also after exposure to the threats posed by a predator (Motta et al., 2009), and entrapped immobilization (Motta and Canteras, 2015). Conversely, passive defensive behavioral responses (typified by “freezing”, and supine posture) associated with exposure to a predator are blocked by cytotoxic lesion of the PMd (Cezario et al., 2008) and PAGd (Sukikara et al., 2010); PMd lesions also abolish passive defensive responses associated with context re-exposure (Cezario et al., 2008). The present data therefore add to, and are consistent with related earlier pathway-tracing, neuroactivational and behavioral data that collectively support a role for LHAjd in the control of defensive behavioral responses.

**Figure 3:**
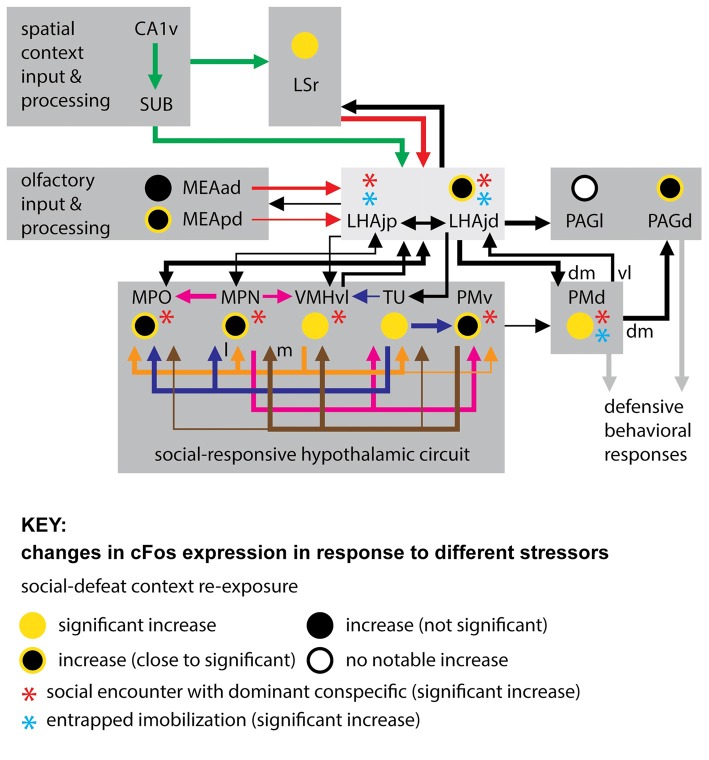
**Connections of the LHAjd and LHAjp with regions indicated to play a key role in the expression of defensive behavioral responses, in particular to socially-relevant threats (stressors).** Also indicated are comparative changes in the levels of cFos expression associated with three different stressors: entrapped immobilization (restraint; Motta and Canteras, 2015), encounter with a dominant conspecific (Motta et al., 2009; Motta and Canteras, 2015), and re-exposure to social defeat-associated context (Faturi et al., 2014; see figure Key for explanation of symbols). The cFos data is shown only for the LHAjd and LHAjp and their connected regions that were included in previous analysis. The connections shown are based on data obtained from previous pathway tracing studies (not all connections are shown): (Simerly and Swanson, 1988; Canteras and Swanson, 1992a; Canteras et al., 1992a, 1994; Risold and Swanson, 1997b; Comoli et al., 2000; Goto et al., 2005; Cenquizca and Swanson, 2007; Motta et al., 2009; Hahn and Swanson, 2010, 2012). Abbreviations: CA1v, Hippocampal region, Field CA1, ventral part; LHAjp, Lateral hypothalamic area, Juxtaparaventricular region; LHAjd, Lateral hypothalamic area, Juxtadorsomedial region; MEAad, Medial amygdalar nucleus, anterodorsal part; MEApd, Medial amygdalar nucleus, posterodorsal part; MPN, Medial preoptic nucleus; MPO, Medial preoptic area; PAGd, Periaqueductal gray, Dorsal division; PAGl, Periaqueductal gray, Lateral division; PMd, Dorsal premammillary nucleus; PMv, Ventral premammillary nucleus; SUB, Subiculum; TU, Tuberal nucleus; VMHvl, Ventromedial hypothalamic nucleus, Ventrolateral part; dm, dorsomedial; l, lateral; m, medial; vl, ventrolateral.

In addition to the LHAjd, PMd and PAGd, several other interconnected gray matter regions (within and outside of hypothalamus) show increased cFos expression in response to social defeat, or in response to re-exposure to the context in which a social defeat occurred (Motta et al., 2009; Motta and Canteras, 2015; those with direct connections to the LHAjd are shown in Figure 3). Five of these regions—the MPO, MPN (embedded within the MPO), VMHvl, TU and PMv—are notable because the available data indicates their increase in cFos expression is striking and significant in response to perceived social threats (Motta et al., 2009; Motta and Canteras, 2015), but not in response to the potentially existential threats of entrapped immobilization (Motta and Canteras, 2015), or predator exposure (Canteras et al., 1997).

Furthermore, the MPO, MPN, VMHvl, TU and PMv, in addition to being implicated by cFos analysis in socially-relevant defensive responses (Motta et al., 2009; Motta and Canteras, 2015), have longer been implicated in other socially-relevant behaviors (for review, see Canteras, 2012). These include aggressive (Kollack-Walker and Newman, 1995; Veening et al., 2005), sexual (Kollack-Walker and Newman, 1995; Veening et al., 2005), and reproductive (Beltramino and Taleisnik, 1985; Risold et al., 1997) behaviors. The MPN and MPO are additionally implicated in maternal (Stack and Numan, 2000), and also paternal (Dulac et al., 2014) behaviors. This plurality of indicated social behavioral roles for the same hypothalamic regions that are themselves highly interconnected lends support to their current inclusion in a conceptual “conspecific/social-responsive hypothalamic circuit” (Motta et al., 2009; Motta and Canteras, 2015; Figure 3). It is noteworthy that a strong association with reproductive behavior and function for this circuit led to its earlier conception as a “medial hypothalamic reproductive system” (Canteras, 2002).

Taking the present results together with the indicated similarities in responses to social defeat, in terms of neuroactivational responses of the LHAjd and the conspecific/social-responsive hypothalamic circuit (Motta et al., 2009; Motta and Canteras, 2015; Figure 3), it suggests that the LHAjd might also participate in other socially-relevant behavioral functions of this circuit. Consistent with this hypothesis, it is noted that the LHAjd has robust connections with the MPO, and receives a moderate input from the VMHvl (Hahn and Swanson, 2012; Figure 3). However, unlike the conspecific/social-responsive hypothalamic circuit, the LHAjd shows a significant increase in cFos expression to entrapped immobilization (Motta and Canteras, 2015; Figure 3), suggesting a wider more general defensive behavior related role.

In support of a possible general role for the LHAjd in defensive behavioral control, in addition to direct connections with the conspecific/social-responsive hypothalamic circuit, the LHAjd also connects directly with a conceptual predator-responsive hypothalamic circuit involving the anterior hypothalamic nucleus (AHN) and the VMH dorsomedial part (VMHdm; see Figure 11 in Motta et al., 2009; Hahn and Swanson, 2012). Furthermore, the LHAjd also connects with regions that appear to be part of an earlier alluded to common pathway for the expression of defensive behaviors in general. Foremost among these regions is the PMd (Blanchard et al., 2003, 2005; Canteras et al., 2008; Cezario et al., 2008), with which the LHAjd has strong bilateral and bidirectional connections (Hahn and Swanson, 2012). The organization of connections between the LHAjd and PMd is noteworthy because while the LHAjd primarily targets a dorsal/dorsomedial PMd subregion, it receives input primarily from a ventral/ventrolateral subregion. This topographic connectional difference has relevance in relation the pattern of PMd cFos occurring in rats in response to social defeat (dorsal/dorsomedial PMd) or predator exposure (ventral/ventrolateral PMd). Thus the LHAjd appears well placed to integrate information relevant to defensive responses to different types of threat.

In the present study an additional and intriguing finding was an increase in active- and decrease in passive defensive responses in LHAjd lesioned intruders during resident-intruder encounters. Although these behavioral changes did not quite reach significance (Table 2), they nevertheless echo a similar yet significant result in the same paradigm after bilateral cytotoxic PMd lesions (Motta et al., 2009). Together these findings not only reinforce a view of the PMd as part of a common pathway for the expression of defensive behavioral responses, but also suggest the LHAjd may be an important upstream component of that pathway. The latter suggestion is also supported by a significant increase in LHAjd cFos occurring in response to entrapped immobilization (Motta and Canteras, 2015). As to why the effects of PMd lesions produce a more pronounced effect than LHAjd lesions, this might be explained by considering a possible contribution of the LHAjp, which is rostrally adjacent to the LHAjd, and has a similar pattern of connections (both input and output), including a dense innervation of the dorsal/dorsomedial PMd (Hahn and Swanson, 2010). Furthermore, the LHAjp also shows a significant increase in cFos expression following social defeat and entrapped immobilization (Motta and Canteras, 2015; Figure 3).

Continuing a consideration of a potentially broad role for the LHAjd in control of defensive behavioral responses, in addition to LHAjd connections with the PMd, and to the previously mentioned PAGd, the LHAjd also has robust downstream connections with several other PAG divisions, notably including the PAG lateral (PAGl) and precommissural (PRC) divisions (Hahn and Swanson, 2012). As noted at the start of this discussion, muscimol blockade of GABA_A_ receptors in the PAGd (and also PMd) in socially defeated rats immediately prior to re-exposing them to the defeat-associated context, results in a significant attenuation of risk assessment behavior (Faturi et al., 2014). Additionally, a robust increase in PAGd cFos expression is seen in these animals after context re-exposure compared to non-defeated controls (Faturi et al., 2014); a similarly robust cFos increase is seen in the PAGl immediately after encounter with a dominant conspecific (Motta et al., 2009). Furthermore, a significant increase in PRC cFos is reported following predator exposure (Canteras and Goto, 1999). However, a specific role for the LHAjd in relation to predator threat remains to be investigated.

Turning from LHAjd downstream connections to those upstream, three sites are prominent: within the striatum the rostral part of the lateral septal nucleus (LSr), and medial nucleus of the amygdala (MEA), and within the cerebral cortex the subiculum (SUB; dorsal and ventral, but mostly its intermediate part—SUBi; see Figure 11 in Hahn and Swanson, 2010, 2012). The MEA is a major recipient of olfactory sensory information, especially behavior-relevant pheromone signals (Swanson and Petrovich, 1998), and in addition to having bidirectional connections with the LHAjd (Hahn and Swanson, 2012), it also provides substantial input to both the conspecific/social-responsive and predator-responsive hypothalamic circuits (Canteras et al., 1995). The major MEA connection with the LHAjd is with the MEA anterodorsal part (MEAad), with a lesser MEA posterodorsal part (MEApd) connection, and weak to very weak connections with the other parts of the MEA (Hahn and Swanson, 2012). All parts of the MEA are interconnected (Canteras et al., 1995), and all show increased cFos expression in socially-defeated rats re-exposed to the defeat context (Faturi et al., 2014); however, in socially-defeated rats the most notable MEA cFos increase is reported to occur in the MEApd and MEAad, in response to the social defeat (Motta et al., 2009), and also in response to re-exposure to the defeat context (Faturi et al., 2014). Furthermore, socially-defeated animals exposed to a similar spatial context but with fresh bedding (i.e., lacking the olfactory cues associated with the aggressor) do not show conditioned defensive responses (Faturi et al., 2014). The present results are consistent with these findings, and with a role for MEA-LHAjd connections in the processing of behaviorally relevant olfactory information.

More broadly, it is worth noting that significantly increased MEAad and MEApd cFos associated with agonistic encounters was previously reported in Syrian hamsters in both dominant and subordinate males, and also following copulation (Kollack-Walker and Newman, 1995). However, while increases of MEA cFos did not differ between dominant and subordinates, a significant difference was reported between the copulatory group (less cFos) and the agonistic encounter group (more cFos), specifically in the caudal MEApd (Kollack-Walker and Newman, 1995). With respect to this difference in neural activation of the MEApd, it is noteworthy that the moderate MEApd connection with the LHAjd and LHAjp is mostly with its rostral half (with little to no connection to the most caudal level of the MEApd; Hahn and Swanson, 2010, 2012). In fact, this correlation is consistent with a model in which differences in the connections of the hypothalamus with the parts of the amygdala that receive input from the accessory olfactory bulb (relaying pheromone signals from the vomeronasal organ) are thought to reflect differences in the genetically-encoded mechanisms that enable different behavioral responses (such as reproductive or defensive) to different olfactory cues (Choi et al., 2005).

In addition to the MEA, the other part of striatum with robust LHAjd connections is the LSr. The LSr in turn receives a major input from the Ammon’s horn and SUB (Risold and Swanson, 1997b), and the latter, in particular the SUBi, provides a major input to the LHAjd (Hahn and Swanson, 2012). The LHAjd also receives an input from the ventral part of CA1; however, this is relatively weak compared to a more substantial ventral CA1 (and ventral SUB) input to the LHAjp (Kishi et al., 2000; Hahn and Swanson, 2010, 2012). The organizational pattern of these connections extends to a general topographic organization between Ammon’s horn and the subiculum, the lateral septal nucleus (LS), and the hypothalamus (Risold and Swanson, 1996, 1997b; Cenquizca and Swanson, 2006, 2007).

Given the close connectional associations between the LSr and LHAjd, and the results of the present study, it is salient to note that socially-defeated rats re-exposed to the defeat context show a significant increase in LSr cFos expression (Faturi et al., 2014). This aligns with earlier data indicating that electrolytic LS lesion reduces behavioral measures of anxiety (Menard and Treit, 1996). Along similar lines, cytotoxic (ibotenic) lesions of the HPF (including ventral CA1 and SUBi) reportedly reduce unconditioned risk assessment behaviors to predator odor cues (an effect not seen with visual cues, or with lesions restricted to the dorsal part of Ammon’s horn and the subiculum; Pentkowski et al., 2006). A consideration of the underlying circuit properties speaks to these effects: the predominant output of the LSr, like other parts of the LS complex (and the striatum in general), is inhibitory (GABAergic; Risold and Swanson, 1997a); whereas hippocampal outputs (like other outputs of the cerebral cortex) are excitatory (glutamatergic; Swanson, 2000). With respect to behavioral responses relevant to defensive behaviors, the connectional relations and differing neurochemistry of the HPF and LS is highlighted by experiments reporting that activation of GABA_A_ receptors in the HPF produces an anxiolytic effect that is blocked by glutamate activation of the LS (Menard and Treit, 2001). However, inclusion of the LHAjd in a model of these behaviorally-relevant circuits should take account not only of the HPF input to the LHAjd, but also of the highly bi-directional (and indicated reciprocal) connections between the LS and LHAjd (Hahn and Swanson, 2012).

A consideration of hippocampal neural connections in defensive behavioral responses was revisited in a recent article that compared and contrasted the pattern of cFos expression in male rats associated with the stressful threats posed by either entrapped immobilization, or an encounter with an aggressive conspecific (Motta and Canteras, 2015). A novel possibility was raised that one role of the subiculum (and LS) to LHAjd (and LHAjp) pathway may be to transmit behaviorally relevant threat-associated spatial boundary information, following recent work further characterizing the role of subicular neurons that respond to environmental boundaries (Stewart et al., 2014). The potential importance of a direct hippocampal to LHAjd (and LHAjp) connection that could integrate spatial information with specific sensory cues to modulate behavioral control was recognized in the first pathway tracing study of the LHAjp: “…one could predict that lesion of the LHAjp may have an effect on spatial (contextual) learning and navigation, and especially in relation to defensive behavior that has an olfactory component.” A similar prediction was subsequently made for the LHAjd: “locational information relayed by hippocampal neurons to the LHAjd could have obvious relevance if the LHAjd is an integral part of a system for the control of defensive behavior.” The present results are consistent with the earlier predictions. More generally, these perspectives inform a growing understanding of regional and topographic hippocampal division of labor with respect to processing and integration of different types of sensory information and the major mnemonic and spatial computation roles of the HPF (Dong et al., 2009; Strange et al., 2014).

As a final point of discussion, and by way of considering possible broader implications of the present results, one might ask how convergent hippocampal-septal and amygdala input to the LHAjd may contribute to the control of defensive behavioral responses? As noted previously, in socially-defeated rats re-exposed to the defeat context, visuospatial context cues alone do not appear sufficient to elicit conditioned defensive responses, which also require the presence of (at least) olfactory cues previously associated with the dominant aggressor (Faturi et al., 2014). Therefore, the LHAjd is in a position to integrate spatially-relevant information (presumably relayed by the subiculum), as well as olfactory-relevant information (presumably relayed by the MEA). In a general sense, the integration of dual sensory input streams conveying threat-relevant information by a hypothalamic region (LHAjd) implicated in behavioral control, may have relevance to (for example) neuropsychiatric diseases in which there is a dysfunction of context-appropriate behavioral responses.

One possible example is post-traumatic stress disorder (PTSD) that is typified by context and cue disassociated (“inappropriate”) defensive (or aggressive) behavior following a traumatic (highly stressful and threatening) event or episode (American Psychiatric Association, 2013). The hippocampus and amygdala are both implicated in PTSD (Shiromani et al., 2009), and an underlying thread common to both appears to be their (indirect) role in, and (direct) responsiveness to, neuroendocrine signaling associated with the stress response—in particular, indirect stress-associated modulation of the hypothalamic-pituitary-adrenal (HPA) axis (Herman et al., 2003), and direct responsiveness to glucocorticoid hormones (Sapolsky et al., 1984; Reul and de Kloet, 1985; Han et al., 2014). It is noteworthy that CA1 and SUB, which provide the single most abundant source of input to the LHAjd (and LHAjp; Hahn and Swanson, 2010, 2012), are the same hippocampal regions that have the highest expression of corticosterone receptors (also highly expressed in the LS nucleus; Reul and de Kloet, 1985); moreover, the expression levels of glucocorticoid receptors in ventral CA1 and SUB is more than double that in the dorsal parts of these regions (Reul and de Kloet, 1985). With regard to the amygdala in relation to PTSD, much attention has been paid to the role of the lateral amygdala in models of fear-conditioning (Debiec and LeDoux, 2009), while the medial amygdala has received less attention. Nevertheless, repeated restraint stress in mice appears to cause a reduction in dendritic spines on MEA neurons (Bennur et al., 2007). More generally, restraint stress and social-defeat in rats both generate a robust increase in cFos expression in the medial parvicellular part of the hypothalamic paraventricular nucleus (indicative of HPA axis activation; Faturi et al., 2014; Motta and Canteras, 2015). Also of broader relevance to the organization of the underlying neural circuits, a substantial input to the amygdala (including a light to moderate input to the MEAad) from the ventral subiculum is noted (Canteras and Swanson, 1992b).

## Conclusion

The present results provide the first direct evidence of a functional role for the LHAjd in the control of socially-relevant defensive behavioral responses, and conditioned context-dependent responses in particular. Additionally, the results are consistent with a previous report indicating increased activation of the LHAjd in the same behavioral model (Faturi et al., 2014). The results are also supported by a more recent study that further suggests the LHAjd and LHAjp may play a broader role in control of behavioral responses to different types of threat (Motta and Canteras, 2015). A key neural circuit node in these behavioral responses is suggested to be the subiculum, which provides a major input to the LHAjd. Future studies will be necessary to determine the role of the LHAjd and subiculum in relation to the existing model, and also in relation to models employing different threat stimuli (stressors). More broadly the results suggest that additional investigations into the role of the LHAjd, and other LHA regions whose connections have been described in recent years (Goto et al., 2005; Hahn and Swanson, 2010, 2012, 2015), may have relevance to a wide range of neuropsychiatric diseases that involve disordered behavioral control.

## Author Contributions

JDH and NSC designed the experiments. JDH and MJR carried out the experiments. MJR did most of the analysis (with additional input from NSC and JDH). MVCB did the statistical analysis. JDH wrote the article (with editorial input from NSC and MJR).

## Conflict of Interest Statement

The authors declare that the research was conducted in the absence of any commercial or financial relationships that could be construed as a potential conflict of interest.

## References

[B1] American Psychiatric Association (2013). Diagnostic and Statistical Manual of Mental Disorders. 5th Edn. Arlington, VA: American Psychiatric Association.

[B2] Beltramino C. Taleisnik S. (1985). Ventral premammillary nuclei mediate pheromonal-induced LH release stimuli in the rat. Neuroendocrinology 41, 119–124. 10.1159/000124164 4047330

[B3] Bennur S. Shankaranarayana Rao B. S. Pawlak R. Strickland S. McEwen B. S. Chattarji S. (2007). Stress-induced spine loss in the medial amygdala is mediated by tissue-plasminogen activator. Neuroscience 144, 8–16. 10.1016/j.neuroscience.2006.08.075 17049177

[B6] Blanchard R. J. Blanchard D. C. Takahashi T. Kelley M. J. (1977). Attack and defensive behaviour in the albino rat. Anim. Behav. 25, 622–634. 10.1016/0003-3472(77)90113-0 562631

[B4] Blanchard D. C. Canteras N. S. Markham C. M. Pentkowski N. S. Blanchard R. J. (2005). Lesions of structures showing FOS expression to cat presentation: effects on responsivity to a Cat, Cat odor and nonpredator threat. Neurosci. Biobehav. Rev. 29, 1243–1253. 10.1016/j.neubiorev.2005.04.019 16084591

[B5] Blanchard D. C. Li C. I. Hubbard D. Markham C. M. Yang M. Takahashi L. K.. (2003). Dorsal premammillary nucleus differentially modulates defensive behaviors induced by different threat stimuli in rats. Neurosci. Lett. 345, 145–148. 10.1016/s0304-3940(03)00415-4 12842277

[B7] Canteras N. S. (2002). The medial hypothalamic defensive system: hodological organization and functional implications. Pharmacol. Biochem. Behav. 71, 481–491. 10.1016/s0091-3057(01)00685-2 11830182

[B8] Canteras N. S. (2012). “Hypothalamic goal-directed behavior—ingestive, reproductive and defensive,” in The Mouse Nervous System, eds Watson C. Paxinos G. Puelles L. (Sydney, NSW: Elsevier), 539–562.

[B12] Canteras N. S. Chiavegatto S. Ribeiro do Valle L. E. Swanson L. W. (1997). Severe reduction of rat defensive behavior to a predator by discrete hypothalamic chemical lesions. Brain Res. Bull. 44, 297–305. 10.1016/s0361-9230(97)00141-x 9323445

[B9] Canteras N. S. Goto M. (1999). Fos-like immunoreactivity in the periaqueductal gray of rats exposed to a natural predator. Neuroreport 10, 413–418. 10.1097/00001756-199902050-00037 10203345

[B13] Canteras N. S. Kroon J. A. Do-Monte F. H. Pavesi E. Carobrez A. P. (2008). Sensing danger through the olfactory system: the role of the hypothalamic dorsal premammillary nucleus. Neurosci. Biobehav. Rev. 32, 1228–1235. 10.1016/j.neubiorev.2008.05.009 18550169

[B14] Canteras N. S. Simerly R. B. Swanson L. W. (1992a). Projections of the ventral premammillary nucleus. J. Comp. Neurol. 324, 195–212. 10.1002/cne.903240205 1430329

[B15] Canteras N. S. Simerly R. B. Swanson L. W. (1992b). Connections of the posterior nucleus of the amygdala. J. Comp. Neurol. 324, 143–179. 10.1002/cne.903240203 1430327

[B16] Canteras N. S. Simerly R. B. Swanson L. W. (1994). Organization of projections from the ventromedial nucleus of the hypothalamus: a Phaseolus vulgaris-leucoagglutinin study in the rat. J. Comp. Neurol. 348, 41–79. 10.1002/cne.903480103 7814684

[B17] Canteras N. S. Simerly R. B. Swanson L. W. (1995). Organization of projections from the medial nucleus of the amygdala: a PHAL study in the rat. J. Comp. Neurol. 360, 213–245. 10.1002/cne.903600203 8522644

[B10] Canteras N. S. Swanson L. W. (1992a). The dorsal premammillary nucleus: an unusual component of the mammillary body. Proc. Natl. Acad. Sci. U S A 89, 10089–10093. 10.1073/pnas.89.21.10089 1279669 PMC50283

[B11] Canteras N. S. Swanson L. W. (1992b). Projections of the ventral subiculum to the amygdala, septum and hypothalamus: a PHAL anterograde tract-tracing study in the rat. J. Comp. Neurol. 324, 180–194. 10.1002/cne.903240204 1430328

[B18] Cenquizca L. A. Swanson L. W. (2006). Analysis of direct hippocampal cortical field CA1 axonal projections to diencephalon in the rat. J. Comp. Neurol. 497, 101–114. 10.1002/cne.20985 16680763 PMC2570652

[B19] Cenquizca L. A. Swanson L. W. (2007). Spatial organization of direct hippocampal field CA1 axonal projections to the rest of the cerebral cortex. Brain Res. Rev. 56, 1–26. 10.1016/j.brainresrev.2007.05.002 17559940 PMC2171036

[B20] Cezario A. F. Ribeiro-Barbosa E. R. Baldo M. V. Canteras N. S. (2008). Hypothalamic sites responding to predator threats–the role of the dorsal premammillary nucleus in unconditioned and conditioned antipredatory defensive behavior. Eur. J. Neurosci. 28, 1003–1015. 10.1111/j.1460-9568.2008.06392.x 18691328

[B21] Chiavegatto S. Scavone C. Canteras N. S. (1998). Nitric oxide synthase activity in the dorsal periaqueductal gray of rats expressing innate fear responses. Neuroreport 9, 571–576. 10.1097/00001756-199803090-00002 9559918

[B22] Choi G. B. Dong H. W. Murphy A. J. Valenzuela D. M. Yancopoulos G. D. Swanson L. W.. (2005). Lhx6 delineates a pathway mediating innate reproductive behaviors from the amygdala to the hypothalamus. Neuron 46, 647–660. 10.1016/j.neuron.2005.04.011 15944132

[B23] Comoli E. Ribeiro-Barbosa E. R. Canteras N. S. (2000). Afferent connections of the dorsal premammillary nucleus. J. Comp. Neurol. 423, 83–98. 10.1002/1096-9861(20000717)423:1<83::aid-cne7>3.0.co;2-3 10861538

[B24] Debiec J. LeDoux J. E. (2009). “Physiology of the amygdala: implications for PTSD,” in Post-Traumatic Stress Disorder, eds Shiromani A. Keane T. M. LeDoux J. E. (New York, NY: Humana Press), 23–48.

[B25] Dong H. W. Swanson L. W. Chen L. Fanselow M. S. Toga A. W. (2009). Genomic-anatomic evidence for distinct functional domains in hippocampal field CA1. Proc. Natl. Acad. Sci. U S A 106, 11794–11799. 10.1073/pnas.0812608106 19561297 PMC2710698

[B26] Dulac C. O’Connell L. A. Wu Z. (2014). Neural control of maternal and paternal behaviors. Science 345, 765–770. 10.1126/science.1253291 25124430 PMC4230532

[B27] Faturi C. B. Rangel, Jr. M. J. Baldo M. V. Canteras N. S. (2014). Functional mapping of the circuits involved in the expression of contextual fear responses in socially defeated animals. Brain Struct. Funct. 219, 931–946. 10.1007/s00429-013-0544-4 23546547

[B29] Goto M. Canteras N. S. Burns G. Swanson L. W. (2005). Projections from the subfornical region of the lateral hypothalamic area. J. Comp. Neurol. 493, 412–438. 10.1002/cne.20764 16261534 PMC2844126

[B28] Goto M. Swanson L. W. (2004). Axonal projections from the parasubthalamic nucleus. J. Comp. Neurol. 469, 581–607. 10.1002/cne.11036 14755537

[B30] Goto M. Swanson L. W. Canteras N. S. (2001). Connections of the nucleus incertus. J. Comp. Neurol. 438, 86–122. 10.1002/cne.1303 11503154

[B31] Hahn J. D. Swanson L. W. (2010). Distinct patterns of neuronal inputs and outputs of the juxtaparaventricular and suprafornical regions of the lateral hypothalamic area in the male rat. Brain Res. Rev. 64, 14–103. 10.1016/j.brainresrev.2010.02.002 20170674 PMC2886810

[B32] Hahn J. D. Swanson L. W. (2012). Connections of the lateral hypothalamic area juxtadorsomedial region in the male rat. J. Comp. Neurol. 520, 1831–1890. 10.1002/cne.23064 22488503 PMC3930178

[B33] Hahn J. D. Swanson L. W. (2015). Connections of the juxtaventromedial region of the lateral hypothalamic area in the male rat. Front. Syst. Neurosci. 9:66. 10.3389/fnsys.2015.00066 26074786 PMC4445319

[B34] Han F. Ding J. Shi Y. (2014). Expression of amygdala mineralocorticoid receptor and glucocorticoid receptor in the single-prolonged stress rats. BMC Neurosci. 15:77. 10.1186/1471-2202-15-77 24947040 PMC4074391

[B35] Herman J. P. Figueiredo H. Mueller N. K. Ulrich-Lai Y. Ostrander M. M. Choi D. C.. (2003). Central mechanisms of stress integration: hierarchical circuitry controlling hypothalamo—pituitary—adrenocortical responsiveness. Front. Neuroendocrinol. 24, 151–180. 10.1016/j.yfrne.2003.07.001 14596810

[B36] Kishi T. Tsumori T. Ono K. Yokota S. Ishino H. Yasui Y. (2000). Topographical organization of projections from the subiculum to the hypothalamus in the rat. J. Comp. Neurol. 419, 205–222. 10.1002/(sici)1096-9861(20000403)419:2<205::aid-cne5>3.0.co;2-0 10722999

[B37] Kollack-Walker S. Newman S. W. (1995). Mating and agonistic behavior produce different patterns of Fos immunolabeling in the male Syrian hamster brain. Neuroscience 66, 721–736. 10.1016/0306-4522(94)00563-k 7644033

[B38] Markham C. M. Blanchard D. C. Canteras N. S. Cuyno C. D. Blanchard R. J. (2004). Modulation of predatory odor processing following lesions to the dorsal premammillary nucleus. Neurosci. Lett. 372, 22–26. 10.1016/j.neulet.2004.09.006 15531081

[B39] Menard J. Treit D. (1996). Lateral and medial septal lesions reduce anxiety in the plus-maze and probe-burying tests. Physiol. Behav. 60, 845–853. 10.1016/0031-9384(96)00138-2 8873261

[B40] Menard J. Treit D. (2001). The anxiolytic effects of intra-hippocampal midazolam are antagonized by intra-septal L-glutamate. Brain Res. 888, 163–166. 10.1016/s0006-8993(00)03046-8 11146063

[B41] Motta S. C. Canteras N. S. (2015). Restraint stress and social defeat: what they have in common. Physiol. Behav. 146, 105–110. 10.1016/j.physbeh.2015.03.017 26066716

[B42] Motta S. C. Goto M. Gouveia F. V. Baldo M. V. Canteras N. S. Swanson L. W. (2009). Dissecting the brain’s fear system reveals the hypothalamus is critical for responding in subordinate conspecific intruders. Proc. Natl. Acad. Sci. U S A 106, 4870–4875. 10.1073/pnas.0900939106 19273843 PMC2660765

[B43] National Research Council, USA (2011). Guide for the Care and Use of Laboratory Animals. 8th Edn. (Washington, DC: National Academies Press), 267–268.

[B44] Pentkowski N. S. Blanchard D. C. Lever C. Litvin Y. Blanchard R. J. (2006). Effects of lesions to the dorsal and ventral hippocampus on defensive behaviors in rats. Eur. J. Neurosci. 23, 2185–2196. 10.1111/j.1460-9568.2006.04754.x 16630065

[B45] Reul J. M. de Kloet E. R. (1985). Two receptor systems for corticosterone in rat brain: microdistribution and differential occupation. Endocrinology 117, 2505–2511. 10.1210/endo-117-6-2505 2998738

[B46] Ribeiro-Barbosa E. R. Canteras N. S. Cezário A. F. Blanchard R. J. Blanchard D. C. (2005). An alternative experimental procedure for studying predator-related defensive responses. Neurosci. Biobehav. Rev. 29, 1255–1263. 10.1016/j.neubiorev.2005.04.006 16120464

[B50] Risold P. Y. Canteras N. S. Swanson L. W. (1994). Organization of projections from the anterior hypothalamic nucleus: a *phaseolusvulgaris*-leukoagglutinin study in the rat. J. Comp. Neurol. 348, 1–40. 10.1002/cne.903480102 7814679

[B47] Risold P. Y. Swanson L. W. (1996). Structural evidence for functional domains in the rat hippocampus. Science 272, 1484–1486. 10.1126/science.272.5267.1484 8633241

[B48] Risold P. Y. Swanson L. W. (1997a). Chemoarchitecture of the rat lateral septal nucleus. Brain Res. Rev. 24, 91–113. 10.1016/s0165-0173(97)00008-8 9385453

[B49] Risold P. Y. Swanson L. W. (1997b). Connections of the rat lateral septal complex. Brain Res. Rev. 24, 115–195. 10.1016/s0165-0173(97)00009-x 9385454

[B51] Risold P. Y. Thompson R. H. Swanson L. W. (1997). The structural organization of connections between hypothalamus and cerebral cortex. Brain Res. Rev. 24, 197–254. 10.1016/s0165-0173(97)00007-6 9385455

[B52] Sapolsky R. M. Krey L. C. McEwen B. S. (1984). Glucocorticoid-sensitive hippocampal neurons are involved in terminating the adrenocortical stress response. Proc. Natl. Acad. Sci. U S A 81, 6174–6177. 10.1073/pnas.81.19.6174 6592609 PMC391882

[B53] Shiromani P. J. LeDoux J. E. Keane T. M. (2009). *Post-traumatic* Stress Disorder: Basic Science and Clinical Practice, (New York, NY: Humana Press), p.xiii, 409.

[B54] Simerly R. B. Swanson L. W. (1988). Projections of the medial preoptic nucleus: a *Phaseolus vulgaris* leucoagglutinin anterograde tract-tracing study in the rat. J. Comp. Neurol. 270, 209–242. 10.1002/cne.902700205 3259955

[B55] Stack E. C. Numan M. (2000). The temporal course of expression of c-Fos and Fos B within the medial preoptic area and other brain regions of postpartum female rats during prolonged mother—young interactions. Behav. Neurosci. 114, 609–622. 10.1037/0735-7044.114.3.609 10883811

[B56] Stewart S. Jeewajee A. Wills T. J. Burgess N. Lever C. (2014). Boundary coding in the rat subiculum. Philos. Trans. R. Soc. Lond. B Biol. Sci. 369:20120514. 10.1098/rstb.2012.0514 24366128 PMC3866438

[B57] Strange B. A. Witter M. P. Lein E. S. Moser E. I. (2014). Functional organization of the hippocampal longitudinal axis. Nat. Rev. Neurosci. 15, 655–669. 10.1038/nrn3785 25234264

[B58] Sukikara M. H. Mota-Ortiz S. R. Baldo M. V. Felicio L. F. Canteras N. S. (2010). The periaqueductal gray and its potential role in maternal behavior inhibition in response to predatory threats. Behav. Brain Res. 209, 226–233. 10.1016/j.bbr.2010.01.048 20138922

[B59] Swanson L. W. (2000). Cerebral hemisphere regulation of motivated behavior. Brain Res. 886, 113–164. 10.1016/S0006-8993(00)02905-X 11119693

[B60] Swanson L. W. (2004). Brain Maps: Structure of the Rat Brain, 3rd Edn. (San Diego, CA: Academic Press), 1–215.

[B61] Swanson L. W. Petrovich G. D. (1998). What is the amygdala? Trends Neurosci. 21, 323–331. 10.1016/S0166-2236(98)01265-X 9720596

[B62] Thor D. H. Flannelly K. J. (1976). Age of intruder and territorial-elicited aggression in male long—evans rats. Behav. Biol. 17, 237–241. 10.1016/s0091-6773(76)90546-0 986812

[B63] Veening J. G. Coolen L. M. de Jong T. R. Joosten H. W. de Boer S. F. Koolhaas J. M.. (2005). Do similar neural systems subserve aggressive and sexual behaviour in male rats? Insights from c-Fos and pharmacological studies. Eur. J. Pharmacol. 526, 226–239. 10.1016/j.ejphar.2005.09.041 16263109

